# Beyond cognitive measures: Empirical evidence supporting holistic medical school admissions practices and professional identity formation

**DOI:** 10.15694/mep.2018.0000274.1

**Published:** 2018-12-05

**Authors:** Sandra Yingling, Yoon Soo Park, Raymond H. Curry, Verna Monson, Jorge Girotti

**Affiliations:** 1 University of Illinois College of Medicine

**Keywords:** situational judgment, assessment, admissions, CASPer, professional identity, Professional Identity Essay, PIE, Defining Issues Test, DIT2, non-cognitive, selection, holistic admission

## Abstract

This article was migrated. The article was marked as recommended.

**Background:** Medical schools seek admissions methods that identify applicants who hold promise to become physicians who will navigate and shape the future medical landscape. The focus on traditional cognitive measures for admission has prompted calls for holistic admissions review during the past five years. Yet, empirical evidence linking selection measures to holistic admissions practices has not been fully established, including their relationship with professional identity formation over time. A non-cognitive admissions situational judgment screening test (CASPer) measuring personal and professional characteristics was added to the University of Illinois College of Medicine admissions process two years ago, as we implemented a new curriculum that emphasizes professional identity development.

**Purpose:** This study examined associations among admissions measures (Medical College Admission Test [MCAT], grade point average [GPA], interview, and CASPer), and their predictive relationships with curricular measures of professional identity formation (Professional Identity Essay [PIE]) and moral reasoning (Defining Issues Test [DIT2]).

**Methods:** Data were taken from two entering cohorts ( *n* = 596; entering class of 2017 and 2018 across 3 regional sites). Correlations and regression analyses were used to examine associations between admissions and professional identity measures.

**Results:** CASPer and in-person admissions interview ratings had significant positive correlations, suggesting that CASPer can contribute to effective screening processes. In addition, CASPer demonstrated statistically significant positive relationships with professional identity (CASPer and PIE, *r*=.10, *p*<.05) and a measure of moral reasoning (CASPer and DIT2 type indicator, *r*=.09, *p*<.05). Association between CASPer and PIE remained consistent, even after controlling for MCAT, interview, and GPA.

**Conclusion:** Our institutional focus on professional identity formation has provided new ways to conceptualize students’ readiness for medical school – demonstrated academic rigor as well as signs of professionalism, ethics, and motivation. Non-academic factors measured in situational judgment tests may promote better alignment of admissions practices and desired educational outcomes.

## Introduction

Historically, medical schools have relied on quantitative, cognitively-based measures like national standardized test scores and grade point averages as the central criteria for medical school admission. However, given the need for physicians to be leaders in addressing complex ethical and moral dilemmas, and in being champions of the health of the communities they serve, medical schools are broadening the scope of their admissions processes. This approach is often referred to as “holistic review,” defined as “a flexible, individualized way of assessing an applicant’s capabilities by which balanced consideration is given to experiences, attributes, and academic metrics.. and, when considered in combination, how the individual might contribute value as a medical student and future physician” ( AAMC, 2013).

Concurrent with the implementation of a newly integrated undergraduate curriculum in 2017, the University of Illinois College of Medicine [hereinafter “the College”] introduced an early and consistent emphasis on professional identity ( Irby and Hamstra, 2016) as the cornerstone of resilience in the face of challenges to professional and ethical behavior. Students complete two measures at the beginning of medical school: the Professional Identity Essay (PIE, Bebeau and Monson, 2012) and a moral reasoning assignment (Defining Issues Test, or DIT2, Rest, 1999; Bebeau, 2002). Individualized written feedback reports and subsequent discussions highlight ways in which students can influence their professional identity formation and increase their capacity for effective, real-time moral reasoning in the clinical workplace.

The College admits approximately 300 students per year and has humanism as its focus: the whole physician serving the whole patient. We thus had a compelling need to respond to the national call for admissions practices that will support our goal of developing physicians who are ready to face complex challenges, to form effective teams, and to turn experiences into knowledge and knowledge into wisdom.We sought evidence-based “non-cognitive” factors in the admissions process that would help to identify strong candidates; this required using selection methods that go beyond standardized testing. The factors that currently determine admissions decisions include academic credentials such as MCAT score and grade-point average; other academic and non-academic experiences in research, clinical settings, campus and community; and personal attributes of the applicant. Those who meet preset criteria are invited to a personal interview, which is added to the previous criteria. The College added an application requirement in 2017: completion of the online Computer-based Assessment for Sampling Personal Characteristics (CASPer). The CASPer data has not been used in admissions decisions to date.

CASPer is an online situational judgment screening test that has demonstrated predictive validity for personal and professional characteristics as many as six years after medical school admission ( Dore, 2017).CASPer was introduced at our College as an additional dimension of non-cognitive characteristics, to supplement the in-person admissions interviews conducted by our administrators, faculty, staff, and students. Admissions interviewers provide ratings that address the professionalism, motivation, and other attributes that each interviewee demonstrates during a 45-minute interview; the ratings are then compiled into a composite interview score for each interviewee.

The College has a commitment to nurture students’ professional identity formation that is reflected in our curriculum. From their first days in medical school, students participate in a structured, longitudinal curriculum of professional identity formation that includes reflective writing and small group discussions led by faculty facilitators. Early in their training, students receive a detailed, personalized feedback report on two measures, the Professional Identity Essay and the Defining Issues Test (DIT2). The feedback report gives students a framework for thinking about how professional identity develops: from first being focused on individual achievement as it is externally defined, to then discerning how to be a contributing member of a clinical team, to then defining for oneself how to take responsibility for complex clinical challenges that have no roadmap and creating one’s own definition of what the community of practice will be.

This study provides empirical support for the inclusion of non-cognitive elements in the admissions process. CASPer is a systematic, efficient, and standardized method of incorporating important evidence of applicants’ orientation toward professionalism, ethics, and motivation that can support and supplement our existing in-person admissions interviews.

## Methods

### Admissions Process

**CASPer as part of admissions process:** CASPer is a situational judgment test that comprises 12 sections, requiring eight responses to video and four responses to written scenarios. CASPer requires applicants to provide open-ended responses rather than choosing from a list of response options. An application is not considered complete without a CASPer score on record. Only those applicants who take the test and submit their score can be considered for potential in-person interviews. This data set is of matriculants whose offer of matriculation was not based on CASPer.

**Interview as part of admissions process:** In-person interviews, average of multiple interviewers including administrators, faculty, staff and students. Results are aggregated into a single 5-point scale.

**MCAT, Science GPA, Cumulative GPA:** All academic data are verified by AMCAS before an application arrives at the college. By July 1 ^st^ (just before enrolling in the college), an admitted student must provide an official transcript that must show that they were awarded a baccalaureate degree.

### Professional Identity Formation in the Curriculum

The College curriculum provides longitudinal integration of themes such as Professional Development, which addresses professional identity formation (PIF) through focused reflective writing and recurring small group discussions with faculty. Professional identity formation is emphasized as a key to understanding and addressing challenges to professionalism and ethical behavior, and is explained within the frameworks proposed by Bebeau and Faber-Langendoen (2014) and Cruess et al. (2014). During medical school orientation, students attend a 90-minute large group introducton to the developmental nature of professionalism, our institution’s role in guiding and supporting the formation of students’ professional identity formation, and each student’s opportunities to prepare and practice and therefore exert influence on their own development. Students receive an assignment to complete both the Professional Identity Essay (PIE) and a moral reasoning measure (Defining Issues Test, DIT2).

At the time the assignment is given, students are provided with the following context: each student will receive a written report that includes individualized feedback, the PIE stage that best reflects their writing, and DIT2 category that best reflects their responses to a set of hypothetical moral dilemmas. The individual written reports are for the students’ use and can be shared or not shared based on the students’ preference; individual reports are not provided to faculty who facilitate PIF small groups. Students are asked to engage fully in the assignment by being thoughtful, by providing complete elaborated responses to prompts, and by being aware that these conceptual frameworks and this report will be referenced throughout the curriculum.

### The Professional Identity Formation Report

Deidentified PIE narrative responses were scored by Dr. Verna Monson and Dr. Aja King. DIT2 responses were scored by the Center for Ethical Development at the University of Alabama. Feedback reports were generated for each student that included a description of professional identity formation stages, the prompts, and the student’s responses, along with individualized narrative feedback with observations and developmental suggestions.

About eight weeks into the curriculum, students receive their PIF reports, gather for a plenary on professional identity formation, and meet with their faculty-facilitated PIF small groups. Students then complete a reflective and analytic writing assignment about their understanding of the PIF report data they received; they are asked to write a note to themselves in the future (“Write to your future self.. What do you want to remind yourself about - your concerns and aspirations as a beginning medical student? What challenges do you anticipate as you begin to act and feel like a physician?”). Their note to themselves is then revisited a year later before they begin clerkships.

### Professional Identity Essay

The Professional Identity Essay (PIE) elicits respondents’ conceptualization of their professional role in society and measures stages of mental complexity (i.e., psycho-social-emotional capacities). PIF stages for individual respondents are assigned based on narrative responses to nine prompts. Previously reported PIE inter-rater ICC: .83, 95% CI [.57 - .96], and intra-rater ICC .85, 95% CI [.50 - .93] (Kalet et al., 2016). The nine prompts to which students are asked to provide thoughtful and fully elaborated responses are:

1. What does being a member of the medical profession mean to you? How did you come to this understanding?
2. What do you expect of yourself as you work towards becoming a full-fledged physician?
3. What will the profession expect of you?
4. What conflicts do you experience or expect to experience between your responsibility to yourself and others—patients, family, and profession? How do you resolve them?
5. What would be the worst thing for you if you failed to live up to the expectations you have set for yourself?
6. What would be the worst thing for you if you failed to live up to the expectations of your patients?
7. What would be the worst thing for you if you failed to live up to what society expects of physicians? How did you come to this understanding?
8. Think of a physician you consider an exemplar of professionalism. Describe why you chose this person, illustrating with an incident or pattern of decisions or actions that supports your choice.
9. Reflect on your experiences in medical school or in the community that have been critical in fostering change in your understanding of what it means to be a professional – to be a physician.

Students’ narrative responses are coded, resulting in descriptions of professional identity that range along a continuum of mental complexity from identity that is primarily externally defined (independent operator and team-oriented idealist) to identity that is increasingly self-defined (self-authoring integrated professional and self-transformational). Four descriptive categories mirror Kegan’s model of developmental stages relevant to adults, Stages 2-5 ( Kegan, 1994). The independent operator roughly equates to Kegan’s instrumental mind (Stage 2), the team oriented idealist to the socialized mind (Stage 3), the self-authoring integrated professional to the self-authoring mind (Stage 4), and self-transformational (Stage 5).

### Defining Issues Test (DIT2)

Multiple studies indicate that medical training itself may decrease sensitivity to moral dilemmas and also may slow growth in moral reasoning (Patenaude et al, 2003; Murrell, 2014). The defining issues test (DIT2) identifies the types of moral arguments an individual finds persuasive when confronted with a moral problem and therefore is a measure of moral judgment, one central component of moral behavior ( Bebeau 2002). The DIT2 presents a series of written cases of moral dilemmas and asks respondents to choose an action from a list, and then to rank a set of justification statements as to how important each statement was in their choice. The resulting DIT2 score reflects the proportion of the time students use universal ethical principles, or defined rules, or personal interest to justify a response to six moral dilemma cases. The DIT2 has been extensively validated and is highly resistant to social desirability bias ( Rest et al, 1999).

An overall score ranging from 1 to 7 is calculated, reflecting the relative proportion of personal interest, maintaining norms, or postconventional thinking that was in evidence (DIT “type” indicator). Separate scores for each of the three categories are also calculated.

### Scoring and Data Analysis

**Data:** We used retrospective data from 596 students collected across two cohorts of the College’s students (cohort 2017: *n* = 286; cohort 2018: *n* = 310). Admissions data collected prior to matriculation (standardized global MCAT scores, undergraduate science and cumulative GPAs, interview scores, and standardized CASPer scores) were merged with professional identity and development data collected during first year of medical school (PIE, DIT: Personal Interest, DIT: Maintain Norms, DIT: Post Conventional, and DIT: Type Indicator).

**Analysis:** Descriptive statistics were used to examine trends across measures and between years. Pearson correlations were used to examine associations. Multiple regression analyses were used to evaluate the predictive relationship between admissions data and professional identity and development data. Data compilation and analyses were conducted using Stata 14 (College Station, TX, USA). The institutional review board at the University of Illinois at Chicago approved this study (Protocol #2012-0783).

## Results/Analysis

### Descriptive Statistics (See Table 1)

Admissions measures (MCAT, Science GPA, Cumulative GPA, interview score, and CASPer) and professional identity and development measures (PIE and DIT measures) were not significantly different between the 2017 and 2018 cohorts. Descriptive statistics pertaining to these measures are presented in Table 1.

**Table 1.** Descriptive Statistics

Factors	Measures	Cohort 2017 ( *n* = 286)		Cohort 2018 ( *n* = 310)		Overall ( *n* = 596)	
Admissions Measures	MCAT	510.17	(7.31)	512.02	(6.13)	511.20	(6.73)
	Science GPA	3.56	(0.33)	3.63	(0.33)	3.60	(0.34)
	Cumulative GPA	3.66	(0.25)	3.72	(0.24)	3.69	(0.25)
	Interview Score	4.26	(0.50)	4.31	(0.44)	4.28	(0.47)
	CASPer	0.12	(0.92)	0.18	(0.91)	0.15	(0.91)
Professional Identity and Development	Professional Identity Essay (PIE)	3.14	(0.42)	3.18	(0.35)	3.16	(0.38)
	DIT: Personal Interest	19.60	(11.26)	18.64	(11.29)	19.10	(11.28)
	DIT: Maintain Norms	25.50	(12.46)	26.69	(13.60)	26.13	(13.07)
	DIT: Post Conventional	50.78	(14.37)	50.48	(14.33)	50.62	(14.33)
	DIT: Type Indicator	6.13	(1.51)	6.10	(1.55)	6.11	(1.53)

Note: Values in parentheses are standard deviations.

### Relationships among Admissions variables (See Table 2)

Cognitive admissions measures (MCAT, Science GPA and cumulative GPA): **Positive linear relationships were found** between MCAT and Science GPA (moderate correlation: r=.36, p<.001), MCAT and cumulative GPA (moderate correlation: r=.32, p<.001), Science GPA and cumulative GPA (strong correlation: r=.94, p<.001).

MCAT and Admissions interview ratings: **No relationship** between standardized admissions test (MCAT) and ratings provided by College admissions interviewers was found (r=-.01, p=.862 NS).

MCAT, Admissions interview ratings and Non-cognitive admissions measure (CASPer): **Positive linear relationships** were found between CASPer and MCAT (modest correlation: r=.12, p<.01) and between CASPer and ratings provided by College admission interviewers (modest correlation: r=.13, p<.01).

### Relationships among Non-Cognitive variables (See Table 2)

**Admissions non-cognitive elements (CASPer and Interview) and measure of professional identity (PIE):** Positive relationships between PIE and CASPer (modest correlation: r=.10, p<.05) and between PIE and Interview (modest correlation: r=.13, p=.002) were found.

**Admissions non-cognitive element (CASPer) and measure of moral reasoning (DIT2):** A positive relationship between DIT2 Type Indicator and CASPer (modest correlation: r=.09, p<.05) was found. **A negative relationship** between DIT2: Personal Interest and CASPer (modest correlation: r=-.09, p<.05) was found.

**Measures of Professional identity formation (PIE) and moral reasoning (DIT2):** Positive relationships between the Professional Identity Essay (PIE) and a measure of moral reasoning (Defining Issues Test, DIT2) were found. PIE and DIT Type Indicator show modest associations: r=.11, p<.05; PIE and DIT Post-Conventional Thinking show a modest correlation: r=.12, p<.01. However, PIE and DIT Personal Interest show a small negative correlation: r=-.13, p

**Table 2.** Associations between Measures: Pearson Correlation

Measure	Statistic	PIE	DIT: Personal Interest	DIT: Maintains Norms	DIT:Post Conventional	DIT: Type Indicator	Casper	MCAT	Science GPA	Cumulative GPA
DIT: Personal Interest	Correlation	–.13								
	*p*-value	.002								
DIT: Maintains Norms	Correlation	–.05	–.31							
	*p*-value	.276	.000							
DIT: Post Conventional	Correlation	.12	–.48	–.63						
	*p*-value	.004	.000	.000						
DIT: Type Indicator	Correlation	.11	–.46	–.38	.72					
	*p*-value	.013	.000	.000	.000					
Casper	Correlation	.10	–.09	.03	.06	.09				
	*p*-value	.017	.034	.508	.176	.043				
MCAT	Correlation	.00	–.12	–.09	.19	.11	.12			
	*p*-value	.943	.005	.039	.000	.008	.004			
Science GPA	Correlation	–.07	–.06	.02	.03	.01	.05	.36		
	*p*-value	.078	.159	.589	.537	.802	.276	.000		
Cumulative GPA	Correlation	–.09	–.05	.00	.05	.03	.07	.32	.94	
	*p*-value	.040	.224	.950	.268	.528	.086	.000	.000	
Interview	Correlation	.13	–.06	–.01	.07	.08	.13	–.01	–.03	–.01
	*p*-value	.002	.119	.777	.100	.057	.002	.862	.466	.870

### Relationships between Admissions variables and Professional Identity Formation variables (See Table 3)

**Predicting PIE:** In a linear regression model, CASPer is consistently a significant predictor variable for PIE scores (p<.05). Admissions interview ratings are also a significant predictor variable for PIE scores (p<.01), but MCAT, science GPA, and cumulative GPA are not.

**Predicting moral reasoning:** In linear regression models predicting various aspects of moral reasoning (DIT2), CASPer is not a significant predictor variable. However, both the MCAT and Admissions Interview ratings are highly significant positive predictors of one of the three moral reasoning schemas: the application of principles, also called “postconventional thinking” (MCAT p<.001 and Interview rating p<.05). MCAT is also a highly significant *negative* predictor of one of the other moral reasoning schemas, maintaining norms (p<.001).

**Table 3.** Relationship between Admissions Measures and Professional Identity Formation: Linear Regression

Measures	Professional Identity Essay		Defining Issues (DIT2)							
			Personal Interest		Maintains Norms		Post Conventional		Type Indicator	
	β	*p*-value	β	*p*-value	β	*p*-value	β	*p*-value	β	*p*-value
Casper	.09	.041 *	–.07	.104	.07	.129	.00	.927	.05	.249
MCAT	–.01	.791	–.08	.091	–.16	.001 **	.22		.12	.008 **
Science GPA	.09	.534	–.09	.498	.21	.130	–.14	.304	–.13	.328
Cumulative GPA	–.19	.180	.12	.393	–.18	.175	.10	.464	.12	.377
Interview	.13	.003 **	–.07	.101	–.04	.388	.09	.034 *	.08	.056

* *p* <.05

** *p* <.01

*** *p* <.001. Effect sizes represent standardized beta coefficients.

## Discussion

As anticipated, the Admissions cognitive measures tended to be highly correlated with each other: MCAT, science GPA, and cumulative GPA all showed significant positive relationships. The MCAT and Admissions Interview ratings displayed no relationship. However, CASPer demonstrated significant positive relationships with both a traditional cognitive measure (MCAT) and with a non-cognitive measure (Admissions interview ratings) as well. Additional sub-analyses of CASPer data will be conducted to determine how CASPer relates conceptually to both the cognitive and non-cognitive measure.

Our newly integrated curriculum incorporates two measures of professional identity formation into our orientation to medical school: the Professional Identity Essay and the Defining Issues Test. We note with interest that CASPer displayed statistically significant positive relationships with both of these PIF measures. Changes in medical students’ professional identity as measured by PIE have been documented during the first 15 months of medical school ( Kalet et al, 2018). Since PIE is based on well-established constructive-developmental theories, further examination of this measure may contribute to the evidence base to guide medical education practice and policy ( Cook, Bordage, & Schmidt, 2008; Rabow et al., 2010).

This study provides empirical support for the inclusion of CASPer in a holistic approach to medical school admissions. CASPer may be a feasible alternative to the more intensive structured in-person admissions interviews, the Multiple Mini-Interviews (MMI; Reiter et al, 2007). The physicians we train need to be context-builders for their community of practice, so we must continue to look beyond standardized test scores and GPA to identify applicants who will grow to become great physicians. Based on two years of data (almost 600 students), CASPer demonstrates significant positive relationships with other admissions measures - and importantly, with some measures used in the curriculum (e.g., stage of professional identity formation). The College curriculum explicitly teaches professional identity formation as a) our institutional responsibility and b) something on which students have more influence if they understand why professional identity formation is important, how it is formed, and how moral reasoning develops through deliberate practice. CASPer is a step toward better alignment of our admissions practices with our educational practices and desired outcomes.

## Conclusion

This study supports the inclusion of CASPer as a contributing non-cognitive element in making holistic admissions decisions. Our institutional focus on professional identity formation has given us new ways to conceptualize individual student differences in readiness for medical school - not just for the academic rigors, but for the life experiences that form the core of the perspective and ethical decision-making required of a professional. These are differences in readiness that, once apparent and explicit, can be embraced as another dimension of our student body’s diversity and strength.

## Take Home Messages

• Applying holistic admissions practices for selecting and recruiting students can contribute to shaping the professional identity of learners.
• Standardized tests of admissions and GPAs are not sensitive to detecting professional identity development.
• Situational judgment tests can be a useful indicator that bridges traditional cognitive measures and non-cognitive measures to form robust admissions signals.
• Measure and monitor professional identity development from early stages of medical education training.

## Notes On Contributors

Sandra Yingling, PhD is the Associate Dean for Educational Planning at the University of Illinois College of Medicine, and Assistant Professor in the Department of Medical Education at the University of Illinois at Chicago. She focuses on curricular innovation, self-regulated learning among medical students, and professional identity formation.

Yoon Soo Park, PhD is Associate Professor in the Department of Medical Education at the University of Illinois at Chicago. His research interests extend across multiple disciplines in psychometrics, biostatistics, educational psychology, and medicine.

Raymond H. Curry, MD is the Senior Associate Dean for Educational Affairs at the University of Illinois College of Medicine, and Professor of Medicine and Medical Education, University of Illinois at Chicago.

Verna Monson, PhD is a consultant to the University of Illinois College of Medicine on the assessment of professional identity formation for medical education. Her research interests include professional education curriculum development and evaluation, and holistic approaches to remediating professionalism lapses.

Jorge Girotti, PhD is the Associate Dean for Admissions and Special Curricular Programs for the University of Illinois College of Medicine, and Assistant Professor in the Department of Medical Education at the University of Illinois at Chicago.

## References

[ref1] Association of American Medical Colleges (2013). Roadmap to excellence: Key concepts for evaluating the impact of medical school holistic admissions. Washington, D.C.

[ref2] Bebeau M. J. (2002). The defining issues test and the four component model: contributions to professional education. J Moral Educ. 31(3),271–295. 10.1080/0305724022000008115 15027443

[ref3] Bebeau M. J.& Faber-Langendoen K. (2014). Remediating lapses in professionalism. Remediation in medical education.(pp.103–127): Springer. 10.1007/978-1-4614-9025-8_7

[ref4] Bebeau M. J.& Monson V. E. (2012). Professional identity formation and transformation across the life span. Learning trajectories, innovation and identity for professional development.(pp.135–162). London: Springer. 10.1007/978-94-007-1724-4_7

[ref5] Cook D. A. Bordage G.& Schmidt H. G. (2008). Description, justification and clarification: a framework for classifying the purposes of research in medical education. Medical Education. 42(2),128–133. 10.1111/j.1365-2923.2007.02974.x 18194162

[ref6] Cruess R. L. Cruess S. R. Boudreau J. D. Snell L.& Steinert Y. (2014). Reframing medical education to support professional identity formation. Academic Medicine. 89(11),1446–1451. 10.1097/ACM.0000000000000427 25054423

[ref7] Dore K. L. (2017). CASPer, an online pre-interview screen for personal/professional characteristics: prediction of national licensure scores|SpringerLink. Advances in Health Sciences Education. 22:327–336. 10.1007/s10459-016-9739-9 27873137

[ref8] Irby D. M.& Hamstra S. J. (2016). Parting the Clouds: Three Professionalism Frameworks in Medical Education. Academic Medicine. 91(12). 10.1097/ACM.0000000000001190 27119331

[ref9] Kalet A. Buckvar-Keltz L. Monson V. Harnik V. Hubbard S. (2018). Professional Identity Formation in medical school: One measure reflects changes during pre-clerkship training. MedEdPublish. 10.15694/mep.2018.0000041.1 PMC10712001 38089226

[ref10] Kalet A. Buckvar-Keltz L. Harnik V. Monson V. Hubbard S. (2017). Measuring professional identity formation early in medical school. Medical Teacher. 39(3),255–261. 10.1080/0142159X.2017.1270437 28033728

[ref11] Kegan R. (1994). In over our heads: The mental demands of modern life. Harvard University Press.

[ref12] Patterson F. Roberts C. Hanson M.D. Hampe W. Eva K. (2018). 2018 Ottawa consensus statement: Selection and recruitment to the healthcare professions. Medical Teacher. Sep 25,1–11. 10.1080/0142159X.2018.1498589 30251906

[ref13] Rabow M. W. Remen R. N. Parmelee D. X. Inui T. S. (2010). Professional formation: extending medicine’s lineage of service into the next century. Academic Medicine. 85(2),310–317. 10.1097/ACM.0b013e3181c887f7 20107361

[ref14] Reiter H. I. Eva K. W. Rosenfeld J. Norman G. R. (2007). Multiple mini-interviews predict clerkship and licensing examination performance. Medical Education. 41:378–384. 10.1111/j.1365-2929.2007.02709.x 17430283

[ref15] Rest J. Narvaez D. Bebeau M. J.& Thoma S. J. (1999). Postconventional moral thinking: A neo-Kohlbergian approach. Mahwah, NJ, US: Lawrence Erlbaum Associates Publishers.

