# Supplementary material for: Beyond cognitive measures: Empirical evidence supporting holistic medical school admissions practices and professional identity formation
Source: MedEdPublish (2016). 2018 Dec 5;7:274. [Version 1] doi: 10.15694/mep.2018.0000274.1 (PMC10711998; doi:10.15694/mep.2018.0000274.1)
Supplement: Peer review history

# Reviewer report: Reviewer response for version 1

**DOI:** 10.21956/mep.19733.r29563

**Published:** 2019-06-08

**Authors:** Barbara Jennings

**Affiliations:** 1 Norwich Medical School

This review has been migrated. The reviewer awarded 4 stars out of 5 This was a very useful paper to read. The literature that was cited, and the concepts explained in the introduction, will be very valuable for academic teams tasked with undergraduate recruitment.I will certainly be recommending it to our admissions team for discussion. The main aim of the research described was to introduce and evaluate the usefulness of a validated online screening test to supplement face to face interviewing, called CASPer.One of the interesting points that the authors explore is how CASPer could contribute to a particular aim. Which was to match recruitment to the needs of the medical profession and to a clearly defined institutional ethos, linked to a stepwise curriculum about professional identity.In the methods section there was a lot to unpack. The study itself was retrospective statistical analysis of associations between variables (of entry criteria and longitudinal performance and attitude measurements).Suggestions and questions for the authors:1. I wondered if the descriptions of the admissions steps and curriculum itself should have been confined to a section of background information (in the introduction or in an appendix). In my opinion, this would have made the research questions and methodology clearer.2. Also, I think a detailed justification of the statistical methods used and any limitations could have been included in the discussion.3. Finally, what do the authors conclude about face to face interviewing. Were there any intrinsic problems noted for inter-rater reliability? Do the authors have any concerns about unconscious bias affecting selection? Could CASPer buffer against this, if so?

Reviewer Expertise:

NA

No decision status is available

# Reviewer report: Reviewer response for version 1

**DOI:** 10.21956/mep.19733.r29561

**Published:** 2018-12-12

**Authors:** Barbara Griffin

**Affiliations:** 1 Macquarie University

This review has been migrated. The reviewer awarded 3 stars out of 5 This paper describes a really interesting process of guiding professional identity formation - the possibilities for later longitudinal analysis of the data are exciting and I encourage the authors to look at trajectories of change across time and whether the rate of change is predicted by entry tests. I would have liked more information on the final CASPer score - how were the 12 sections combined for example? A key issue that remains unanswered relates to the construct validity of both CASPer and the MMI. Until there is evidence of what specific non cognitive quality they are assessing, it is difficult to know how to best use them in selection. Given the very low correlations in this study, one must conclude they are assessing different qualities, and there is always the concern that this level of association may not be practically relevant.

Reviewer Expertise:

NA

No decision status is available

# Reviewer report: Reviewer response for version 1

**DOI:** 10.21956/mep.19733.r29562

**Published:** 2018-12-10

**Authors:** Linda Nield

**Affiliations:** 1 West Virginia University School of Medicine

This review has been migrated. The reviewer awarded 4 stars out of 5 Thank-you, Dr. Yingling and Colleagues:I found this work very well written and helpful to my role as Admissions Dean. It provides more evidence to consider making a situational judgement test, like CASPer, to be a required part of our holistic review of applicants. One minor suggestion for improvement: in Table 1, please indicate if the values listed (GPA, MCAT, etc) are the means of the applicants or another measure?

Reviewer Expertise:

NA

No decision status is available
